# Digestive tract hemorrhage due to complications with gastrointestinal stromal tumor treated with sunitinib: A case report

**DOI:** 10.3892/ol.2012.1050

**Published:** 2012-11-28

**Authors:** YAN LIU, HE-LONG ZHANG, YE ZHANG, JIA-ZHUAN MEI, HONG-WEI LIN, YAN-WEI GUO, RUI-JUN LI, XIANG-RUI MENG, GUI-JU LIU, MIN LI, PENG XIAO, HUA BAI

**Affiliations:** 1 Department of Oncology, Zhengzhou People’s Hospital, Southern Medical University, Zhengzhou, Henan 450003;; 2 Department of Oncology; Tangdu Hospital, Fourth Military Medical University, Xi’an, Shaanxi 710038, P.R. China; 3 Center of Diagnosis and Treatment for Infectious Diseases, Tangdu Hospital, Fourth Military Medical University, Xi’an, Shaanxi 710038, P.R. China

**Keywords:** gastrointestinal stromal tumor, sunitinib, hemorrhage

## Abstract

Gastrointestinal stromal tumors (GISTs) are rare, and account for 1% of all gastrointestinal neoplasms. GISTs are the most frequent mesenchymal tumors of the gastrointestinal tract. However, the clinical and pathological characteristics of these neoplasms are not adequately understood. The best treatment approach for GISTs remains unclear. In the present study, we report a case of a GIST originating from the stomach. A digestive tract hemorrhage occurred as a complication of sunitinib treatment. This is the first report of a digestive tract hemorrhage due to sunitinib treatment.

## Introduction

Gastrointestinal stromal tumors (GISTs) are a type of cancer that develops in supportive or connective tissues of the digestive system (1). The disease generally affects adults aged 50–70 years, but gender predilection is unclear. The most frequent site of occurrence is the stomach (60% of cases), followed by the small bowel (35%) and other sites (colon, rectum and esophagus; <5%) (2). They primarily arise from mesenchymal tumors of the gastrointestinal tract. Previous evidence demonstrated that most GISTs originate from Cajal pacemaker cells; however, the presence of receptors in omental, mesentery and uterine tumors has raised doubts about the exclusivity of their origin from pacemaker cells (3–5). GISTs express the cell surface transmembrane receptor KIT, which leads to uncontrolled cell proliferation and resistance to apoptosis upon activation (6–9). Tumor resection is one option for treating the localized disease, but recurrence is common. Tyrosine kinase inhibitors (TKIs) such as imatinib and sunitinib are the standard therapy for metastatic or unresectable GISTs (10,11). Usually, Response Evaluation Criteria in Solid Tumors (RECIST) combined with imaging data (CT scan and PET) are used to assess tumor response to treatment (12,13).

## Case report

**Figure 1.**
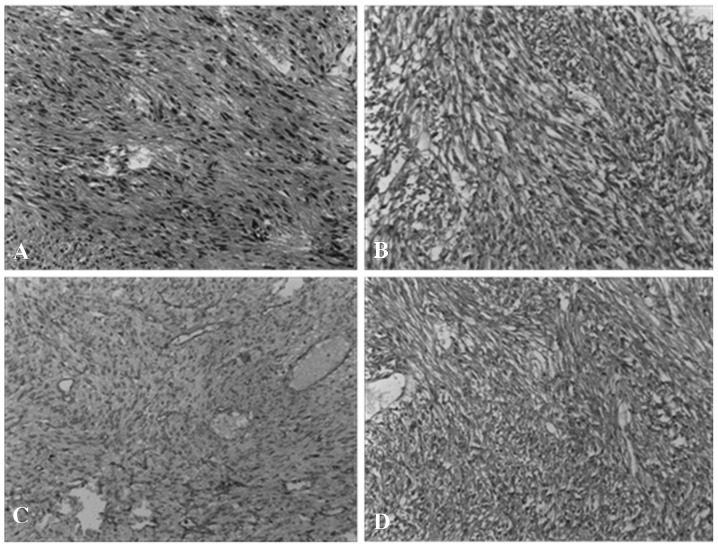
(A) Hematoxylin and eosin staining of gastrointestinal stromal tumors (GIST). The immunohistochemistry study of the GIST revealed positive results for CD117 (B), CD34 (C) and DOG-1 (D).

**Figure 2.**
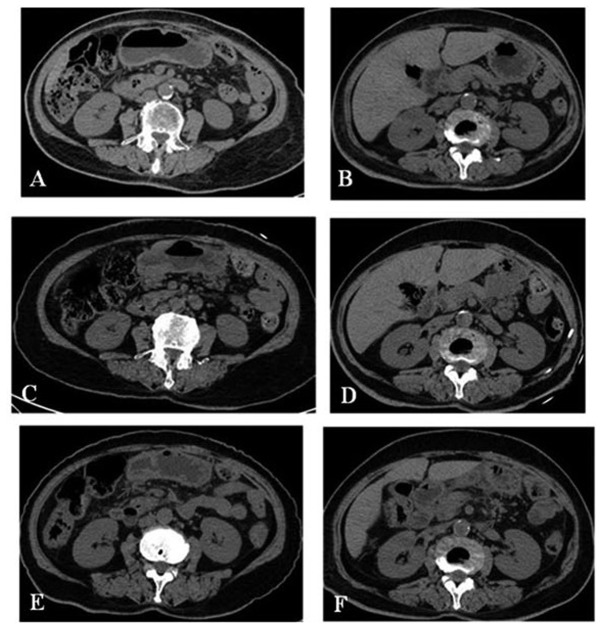
(A) Stomach CT following surgery. (B) CT demonstrated no lymph node metastasis in the abdominal cavity. (C) Stomach CT showed recurrence. (D) CT demonstrated lymph node metastasis in the abdominal cavity. (E) Stomach CT showed that the mass was reducing in size. (F) CT demonstrated that the abdominal cavity lymph node was reducing in size.

An 80-year-old female underwent several examinations in August 2009 for a gall stone. A CT scan disclosed a gastric mass. The patient underwent a partial gastric resection in September 2009 (Fig. 1A). The tumor size was 7.5×5 cm, and the immunohistochemical analysis revealed the tumor was positive for CD117 (Fig. 1B), CD34 (Fig. 1C) and DOG-1 (Fig. 1D), but negative for S100. The patient started imatinib treatment at 400 mg/day and was examined every three months (Fig. 2A and B). She remained well, and stopped imatinib treatment in March 2011. In June 2011, when the patient was referred to Zhengzhou People’s Hospital, recurrence was documented in the gastric remnant (Fig. 2C and D). Beginning in July 2011, she was treated with sunitinib (37.5 mg/day), but demonstrated poor tolerance. She experienced frequent lack of hunger, fatigue, somnolence, nausea and vomiting. In August 2011, she was hospitalized for fatigue. A CT scan presented reductions in the size of the gastric mass and enlarged lymph nodes (Fig. 2E and F). In August 2011, the patient began to exhibit hematemesis and was hospitalized. Later, she presented with digestive tract hemorrhage, and following this, melena and bloody stool occurred. On September 4, 2011, the patient’s hemoglobin concentration was 102 g/l. By September 6, 2011, the hemoglobin concentration was down to 76 g/l. Therefore, conservative medical management was adopted. Hemorrhage stopped gradually. Although the patient experienced gastrointestinal bleeding complications, her treatment was effective. Thus, we suggested continuing sunitinib treatment at a reduced dose or participating in clinical trials of new drugs. The patient rejected these suggestions. She is currently receiving best supportive care (BSC), and follow-up is in progress. Written informed consent was obtained from the patient for publication of this case report and accompanying images.

## Discussion

Pathogenetic mechanisms of GISTs are poorly understood. KIT and PDGFRA mutations drive mesenchymal tumors, including GISTs (gastrointestinal tract sarcomas). Histologically, GISTs vary from spindled to epitheloid and mixed cell tumors. The pathological features are different according to different sites. Gastric GISTs appear as spindle cells and epitheloid cells, but most small intestinal GISTs are spindle cells. Mutations in KIT or PDGFRA lead to increased cellular proliferation and decreased apoptosis. Approximately 85% of GISTs have mutations in KIT or PDGFRA (14–17). Tumors with kinase mutations in exon 11 or 9 have a higher overall response to therapy with receptor tyrosine kinase; therefore, these patients have a significantly longer overall survival.

GISTs are often presented with related symptoms such as anemia or mucosal ulcerations. The diagnostic evaluation is determined by pathological examination. KIT (CD117) is a transmembrane receptor which is a part of the tyrosine kinase receptor complex. GISTs are typically immunoreactive for KIT, thus the presence of CD117 confirms GIST diagnosis by immunohistochemistry. Approximately 90–100% of GISTs express CD117, and 70–80% are positive for CD34, which is the hematopoietic progenitor cell antigen (3,18,19).

GISTs are not sensitive to conventional chemotherapy. The response rate to chemotherapy is <10%. However, targeted therapy has shown some promising results. Imatinib mesylate (a TKI) is considered to be the standard first-line agent in the treatment of unresectable or metastatic GISTs (20). Imatinib, formerly known as STI-571, has been shown to decrease the density of tumor cells without causing inflammation or necrosis (21–23). Sunitinib is an oral multi-targeted tyrosine kinase inhibitor with activity against KIT, PDGFRs, VEGFRs, glial cell line-derived neurotrophic factor receptor, colony-stimulating factor 1 receptor (CSF-1R) and FMS-like tyrosine kinase-3 receptor (FLT3) (24–29). Sunitinib appears to be an effective treatment for patients with imatinib-resistant/intolerant GISTs (7).

TKI-associated side effects mainly include nonhematological and hematological toxicities (30). TKI-associated side-effects affect the curative effect. Thus, the appropriate management of TKI-associated side-effects is important. However, systematic research on the management of TKI-related toxicities remains scarce.

In conclusion, the complication of digestive tract hemorrhage in patients treated with sunitinib is rare. However, this case demonstrates that it does occur. Thus, we should be watchful of this complication in the clinic with sunitinib treatment. Its mechanism remains unclear, therefore data on molecular background, risk factors, treatment response and prognostic significance should be collected in a larger patient population and be further defined.
